# 2041. Impact of Coronavirus Disease 2019 pandemic on healthcare-associated infections at intensive care unit in South Korea: Data from Korean National Healthcare-associated Infections Surveillance System (KONIS)

**DOI:** 10.1093/ofid/ofac492.1663

**Published:** 2022-12-15

**Authors:** Yu-Mi Lee, Dong Youn Kim, Eun Jin Kim, Ki-Ho Park, Mi Suk Lee

**Affiliations:** Kyung Hee University Hospital, Seoul, Seoul-t'ukpyolsi, Republic of Korea; Division of Infectious Diseases, Department of Internal Medicine, Kyung Hee University Hospital, Kyung Hee University School of Medicine, Seoul, Republic of Korea, Seoul, Seoul-t'ukpyolsi, Republic of Korea; Ajou University School of Medicine, Suwon, Kyonggi-do, Republic of Korea; Kyung Hee University Hospital, Seoul, Seoul-t'ukpyolsi, Republic of Korea; Division of Infectious Diseases, Department of Internal Medicine, Kyung Hee University Hospital, Kyung Hee University School of Medicine, Seoul, Seoul-t'ukpyolsi, Republic of Korea

## Abstract

**Background:**

Coronavirus Disease 2019 (COVID-19) pandemic has influenced hospital infection control practices. We evaluated the impact of the COVID-19 pandemic on healthcare-associated infections (HAIs) in the intensive care unit.

**Methods:**

We conducted a retrospective analysis using data from the Korean National Healthcare-Associated Infections Surveillance System (KONIS). KONIS has conducted nationwide prospective surveillance of HAIs in intensive care units. Comparisons of incidence rates of bloodstream infection (BSI), central line-associated bloodstream infection (CLABSI), catheter-associated urinary tract infection (CAUTI), and ventilator-associated pneumonia (VAP) before (Jan 2018-Dec 2019) and during the COVID-19 pandemic (Jan 2020-Jun 2021) were performed according to hospital size. The microorganism distributions in BSI, urinary tract infection, and pneumonia were compared between the period before and during the COVID-19 pandemic.

**Results:**

The incidence rate of BSI significantly decreased during the COVID-19 pandemic than in the pre-COVID-19 period (1.38 vs. 1.19 per 10,000 patient-days; *P* < 0.001) [Table 1]. Incidence rates of CLABSI (2.30 vs. 2.17 per 1,000 device-days; *P* = 0.03) and VAP (1.03 vs. 0.81 per 1,000 device-days; *P* < 0.001) decreased significantly during the COVID-19 pandemic than in the pre-COVID-19 period, whereas that rate of CAUTI was similar between the two periods. The annual trends of incidence rate of CLABSI and VAP also has decreased (Figure 1). According to the hospital size, the incidence rate per 1,000 device-days of CLABSI significantly decreased during the COVID-19 pandemic than in the pre-COVID-19 period in small to medium-sized hospitals (300-699 beds). The incidence rate per 1,000 device-days of VAP significantly decreased only in small-sized hospitals (200-449 beds). The microorganism distributions in HAIs did not change significantly.

**Figure:**
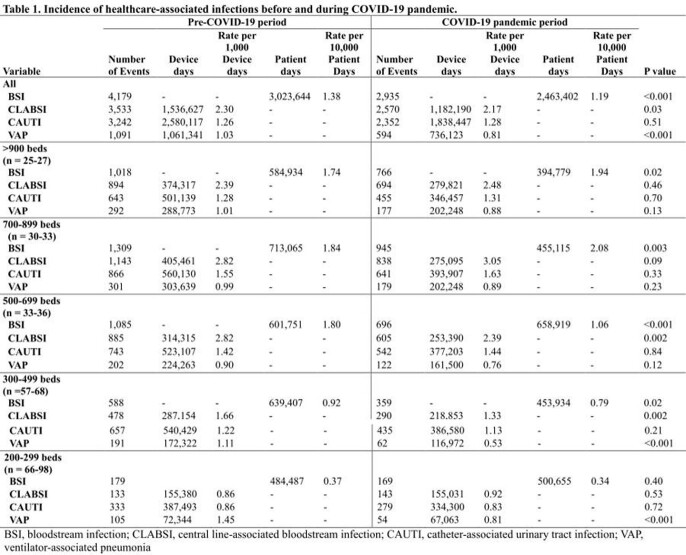

**Figure:**
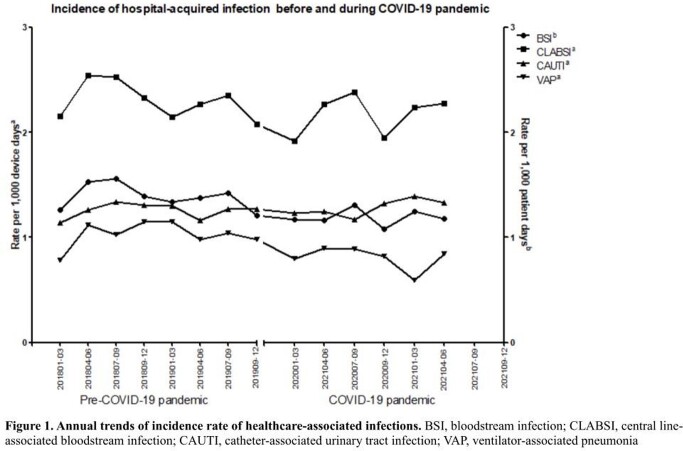

**Conclusion:**

The incidence rates of CLABSI and VAP decreased during the COVID-19 pandemic than in the pre-COVID-19 period, which was attributable to the changes in small to medium-sized hospitals.

**Disclosures:**

**All Authors**: No reported disclosures.

